# The Combined Effect of Glass Fiber Mesh and Steel Fiber on Two-Layered Preplaced Aggregate Concrete against Drop Weight Impact

**DOI:** 10.3390/ma15165648

**Published:** 2022-08-17

**Authors:** Muniraj Karthikeyan, Maruthasalam Verapathran, Sallal R. Abid, Gunasekaran Murali

**Affiliations:** 1 Department of Civil Engineering, Vignan’s Foundation for Science Technology and Research, Guntur 522213, India; 2 Department of Civil Engineering, Dr. N.G.P. Institute of Technology, Coimbatore 641048, India; 3 Department of Civil Engineering, Wasit University, Kut 52003, Iraq; 4 Peter the Great St. Petersburg Polytechnic University, 195251 Saint Petersburg, Russia; 5 Division of Research & Innovation, Uttaranchal University, Dehradun 248007, India

**Keywords:** concrete, steel fibers, glass fiber mesh, impact energy, failure, grout injection

## Abstract

Buildings and other infrastructure should be designed to withstand impact loads in terrorist attacks or industrial catastrophes. Fibrous concrete is utilized in a variety of ways in the construction of structures to resist impact loads. Preplaced aggregate fibrous concrete (PAFC) has a different production method than traditional fibrous concrete. Although PAFC offers several benefits over standard fibers in the construction of protective structures, there are relatively few investigations on the behavior of PAFC when exposed to impact loads. This research investigated the impact response of PFAC with the combined action of steel fibers and glass fiber mesh (GFM). Twenty-two mixtures were prepared and divided into two groups in which there were fibrous and non-fibrous specimens. The specimens from the first group comprised various diameters (ratio of reinforcement surface to total sample surface) of GFM and were provided in two and four layers. The second group of specimens is the same as the first group, including a 3% dosage of steel fibers. All specimens were subjected to a drop-weight impact test and the key parameters examined were cracking and failure impact energies, ductility index and failure pattern. The results indicated that the incorporation of GFM increased the impact performance and impact ductility, where the retained impact energies and the ductility index increased by increasing the ratio of reinforcement surface (diameter) of GFM and its number of layers. However, the positive influence of SF in enhancing the impact performance was way higher than that of GFM. The use of 3% hooked-end SF improved the failure impact energy by more than 3000%, while the maximum improvement gained by using four layers of 150 mm diameter (full reinforcement surface) GFM was approximately 400%.

## 1. Introduction

Modern social and economic progress often necessitates designing and building structures [1,2,3] that can endure catastrophic events like explosions, aircraft strikes, storms and earthquakes and do so with minimal feasible destruction [4]. New materials like Preplaced Aggregate Fibrous Concrete (PAFC) have been developed due to this predicament. A unique kind of concrete known as PAFC is produced by a casting process distinct from the one used to produce traditional fibrous concrete [5]. In PAFC, the formwork is first packed with the required quantity of coarse aggregate and the previously determined amount of fibers. After that, the spaces between the aggregates and fibers are closed with a grout that is free to flow [6]. Different countries use a variety of names to refer to preplaced aggregate concrete (PAC), such as rock-filled concrete, injected aggregate concrete, two-stage concrete, grouted aggregate concrete, prepacked concrete, Arbeton, Naturbeton, PolCrete and Colcrete [7,8,9]. There are two common ways to carry out PAC grouting: gravity or pumping method [10]. In the gravity method, the grout is poured on top of the placed aggregate and lets the grout flow down to the bottom under gravity. During the pumping process, the grout is introduced into the aggregate mass from the base up via a series of pipelines. Based on previous research [11,12], it was concluded that the mechanical characteristics of PAC are influenced mainly by the coarse aggregate qualities and the grout mixture percentage. Nevertheless, there is currently limited publicly available information on PAFC.

Steel fibers have been employed for several decades in several concrete applications, such as in different precast elements, tunnel linings, bridge decks, and pavements [13,14]. Concrete that contains steel fibers may decrease fracture formation, reduce crack breadth and restrict its extension through a crack-bridging process, which has been extensively studied. Additionally, steel fibers improve the strength of concrete by making it more difficult for the fibers to be de-bonded and pulled out of the matrix phase [15]. Cementitious matrix characteristics, a dosage of fiber, aspect ratio, length and shape all affect the engineering characteristics of steel fiber-based concrete [16]. According to the earlier study [17], using steel fiber at doses of up to 1% enhances the concrete splitting and flexural strengths substantially while having little to no influence on the concrete’s ability to withstand compression. Including a higher dosage of steel fiber might negatively influence the concrete’s compressive and tensile strengths [18]. In traditional steel fibrous concrete, the steel fiber dose is typically capped at 2% owing to workability concerns and in an effort to preserve an even distribution of steel fibers throughout the composite. Therefore, greater steel fiber doses (that is, more than 2%) have a tendency to develop fiber balling and clustering, which induces voids and causes weaknesses and faults where micro-cracks may begin, ultimately resulting in a reduction in compressive strength. [19], as a result of which concrete’s mechanical characteristics are jeopardized by an increase in voids [20]. Because of this, the ACI 544.1 [21] established a maximum practicable dosage of steel fiber in traditional concrete of 1.5–2%, which could only be achieved by regular mixing and laying methods. More content of fibers during mixing creates the balling effect and leads to the non-uniform distribution of fibers. The PAFC manufacturing technique, on the other hand, avoids these aforementioned issues since the coarse aggregates and fibers are inserted before the grout is injected. As a result, it is intriguing that greater PAFC compressive strength values were obtained with steel fiber doses up to 6% [5].

Only a limited number of studies have been employed to study the mechanical properties of PAFC. Nehdi et al. [5] reported that the PAFCs’ compressive and tensile properties improved when the dosage of steel fiber was increased. In contrast, the steel fiber length in PAFC had a marginal impact on the compressive and tensile strengths. Steel fiber addition significantly improved both the flexural strength and the post-crack behavior of PAFC. The best post-crack behavior was obtained with PAFC specimens containing 6% of the long steel fiber. The highest toughness index values were achieved for PAFC specimens with a steel fiber dosage of 6%. Mohammadhosseini et al. [22] examined the strength and transportability of a PAFC reinforced with waste polypropylene carpet fibers. Some of the cement was replaced with ash derived from palm oil burning. The gravity technique was used to create six different PAFC mixtures, each with fibers ranging in percentage from 0 to 1.25% and having a length of 30 mm. The grout was injected into the spaces between the aggregates using the pumping technique for six batches, each of which had the same amount of fiber in the same proportions as the previous batch. Results indicated that the compressive strength of PAFC materials supplemented with fibers decreased, and substantial improvements in the splitting tensile strength were reached for fiber dosages up to 0.75% for both the gravity and pumping groups. Nandhu Prasad and Murali [23] reported that the number of hits causing the initial crack and failure of steel fiber-based PAFC enhanced by about 844 and 2700%, respectively, compared to reference specimens without fiber. These impact numbers were increased by about 444% and 588%, respectively, in the case of PAFC comprising 2.5% dosage of polypropylene fiber. Murali and Ramprasad [24] evaluated the impact behaviour of TSFC slabs under dropping mass impact. The three-layered slab, top, middle and bottom layers were reinforced with varying fiber doses of 4%, 2% and 4%, respectively. The results demonstrate that layered TSFC had a better impact energy adsorption capability, eradicated its brittleness and delayed the crack formation and extension.

Many civil engineers have tried to include GFM into cementitious composites due to its recent technological advancements to attain desired properties. Rithanyaa et al. [25] investigated three-layer PAFC slabs against the falling mass impact. The dosage of steel fiber used in the outer layers was 3% and 1.5% for a middle layer with GFM insertion. Findings revealed that the action of the GFM and steel fibers interrupted fracture development, demonstrating superior absorbing impact energy and delaying crack growth. Abirami et al. [26] examined the influence of glass fiber mesh on three-layered TSFC subject to falling mass impact. The glass fiber mesh of various diameters ranging from 50 to 150 mm were inserted from 1 to 4 numbers at the top layer and 4 to 1 number at the bottom layers and vice versa. The TSFC was made with a 2.5% hooked-end steel fiber dosage. Results showed that the glass fiber mesh diameter in TSFC was increased, resulting in a higher increase in impact energy. When comparing the TSFC specimens with 50 mm, 75 mm, 100 mm and 125 mm diameter glass fiber mesh, the specimen with 150 mm diameter glass fiber mesh showed the highest impact energy by approximately 43.5%, 34.3%, 18.3% and 7.2%, respectively. The GFM addition in engineering cementitious composite was studied by Pan et al. [27], who also examined the fatigue performance of the material for use in runway applications. Conclusions revealed that the GFM-reinforced composite can withstand 30,000 blows without incurring any degradation and can take 800 times more strikes before cracking than an ordinary concrete pavement.

There is a dearth of knowledge about the impact performance of PAFC specimens that include steel fibers in addition to GFM sandwiched between the layers of the specimens. Due to this research gap, academicians have turned their focus to this cutting-edge area of research that is now receiving much attention. Unfortunately, there is not a lot of published research accessible in this field; thus, there is a need for further focus in this area. The impact performance of layered PAFC is currently unknown and requires more investigation. Consequently, this research aimed to understand the three layers that make up the PAFC, including the GFM sandwiched between the layers and the steel fibers. Additionally, the effect of the ratio of reinforcement surface to total sample surface of GFM (50 mm, 75 mm, 100 mm, 125 mm and 150 mm diameter with two and four layers) in PAFC is studied against the impact loading.

## 2. Materials and Methods

### 2.1. Base Materials

**Figure 1:**
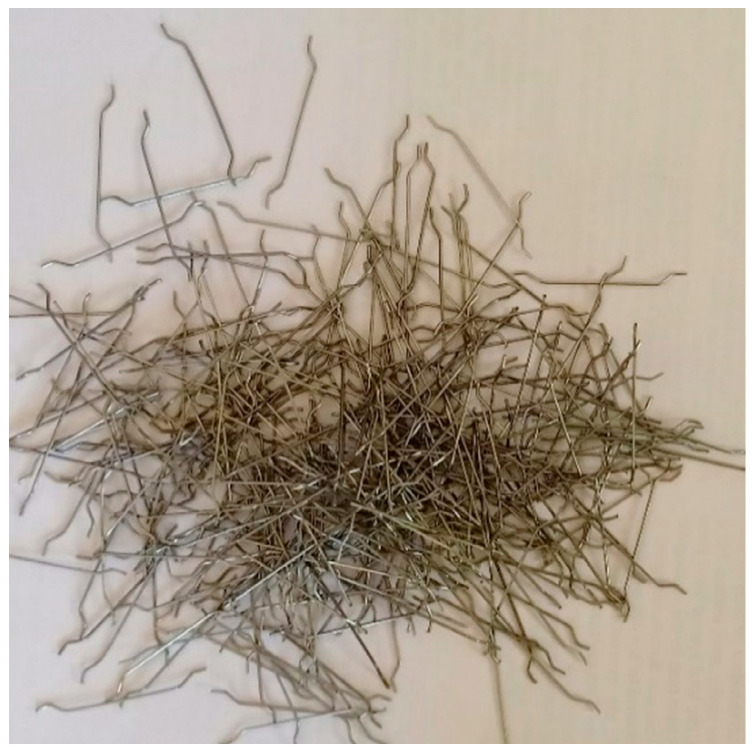
Hooked-end fiber.

**Figure 2:**
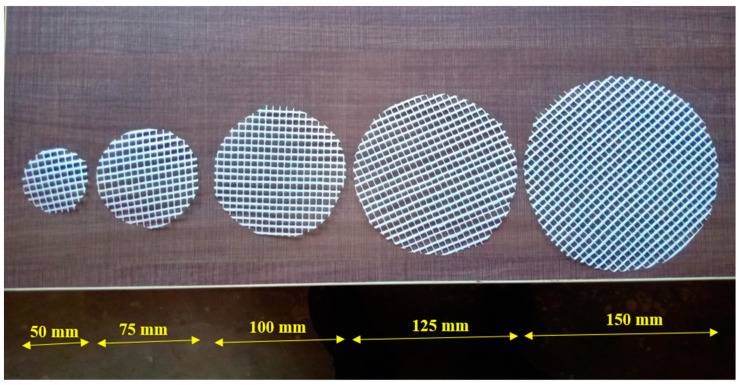
GFM with varying diameters.

- Ordinary Portland Cement was utilized in this investigation as per the specifications outlined in Indian Standard IS: 12269–1987 [28]. Its specific gravity was 3.14 and had a specific surface area of 318 kg/m^2^.
- As per the requirements of the standard IS: 383–2016 [29], the fine aggregate was obtained from a natural river locally. It had a specific gravity of 2.65 and a fineness modulus of 2.41. Additionally, a unique grout mix in accordance with ASTM C939/C939M-16a [30] was confirmed by using the size of fine aggregate particles less than 2.36 mm. After that, a great gravity flow was accomplished, which resulted in the voids inside the skeletal aggregate being effectively filled.
- The dimension of the granite gravel that was employed for the coarse aggregate was 12.5 mm in size. The coarse aggregate had a bulk density of 1700 kg/m^3^, water absorption value of 0.56% and specific gravity of 2.69.
- A superplasticizer (Tec Mix 640) was utilized to enhance the grout flowability and meet the efflux time requirements. The used superplasticizer had a pH value of 7–8. The two dosages of selected superplasticizers were 0.4% and 0.6% (by cement weight) for the non-fibrous and fibrous specimens, respectively. PAFC is the fibrous mixture, and a higher superplasticizer dosage is required to make the grout flow under gravity. However, more flowable grout is required to fill the voids between the fibers and aggregates in a fibrous mixture. Hence, the superplasticizer dosage is enhanced in PAFC
- Hooked-end steel fiber, which has a 30 m length and 0.5 mm diameter, was employed in the concrete at a 3% dose. Figure 1 shows the steel fiber used and its tensile strength was 1400 MPa.
- Grids of two-way glass fiber reinforcement spaced at 5 mm intervals, 2 mm thickness and weighing 125 g/m^2^ per unit area were used in this study. The tensile strength of GFM was 25 KN/m. The GFM roll was sourced from Amazon India, and was cut for a circular shape of different diameters, as shown in Figure 2. The ratios of reinforcement surface to total sample surface were 0.33, 0.5, 0.67, 0.83 and 1.0.

### 2.2. Mixing Combination and Specimens Id

**Figure 3:**
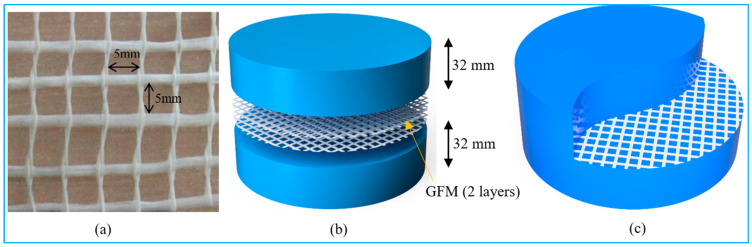
Size of GFM and location of insertion (**a**) square grid of GFM, (**b**) GFM location from the top and bottom surface, (**c**) appearance of GFM after insertion.

This experiment created a total of twenty-two different combinations, each of which had the same proportions of cement to sand (*c*/*s*) and water to cement (*w*/*c*), respectively. Many trial grout mixes were generated to optimize the *w*/*c* and *c*/*s* ratios and the superplasticizer dosage required to match the efflux time of 35–40 s [30]. Among twenty-two mixtures, the first mixture was used as a reference specimen with no fibers and GFM. Another reference mixture is FC, comprising a 3% dosage of steel fiber. The remaining twenty mixtures were divided into two groups. The specimens from the first group comprised various diameters (50 mm, 75 mm, 100 mm, 125 mm and 150 mm) of GFM and were sandwiched in two and four layers between the layered PAFC. The GFM pattern for the second group of specimens is the same as the first group, including a 3% dosage of steel fibers. The mixtures id G2-50 denote the two layers of GFM with 50 mm diameter. Likewise, FG2-50 denotes the specimen comprising a fiber and two layers of 50 mm diameter of GFM. Similarly, the remaining group of specimens was named according to the diameter of GFM and the presence of fibers. Table 1 demonstrates the mixture id of all specimens and mixing combinations. The GFM was sandwiched at a depth of 32 mm from the bottom or top of the specimen. Figure 3 shows the location of the sample GFM sandwiched in the PAFC specimen. Two and four grid plates of GFM were inserted between the two PAFC layers.

### 2.3. Preparation of Specimens

**Figure 4:**
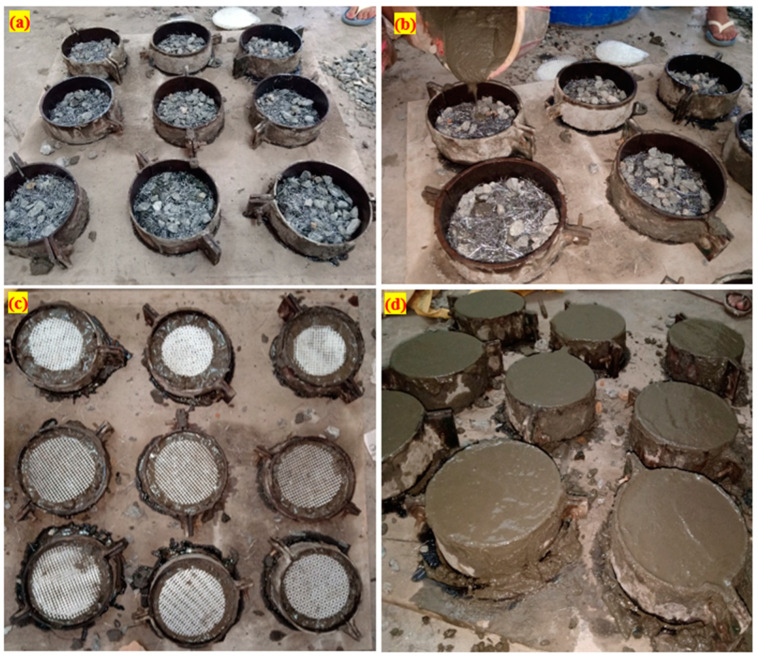
PAFC casting procedure (**a**) mold filled with aggregates and fibers, (**b**) grout injection, (**c**) GFM placement, (**d**) finished specimens.

In order to measure the PAFCs’ resistance to impact, a cylindrical disc specimen was made and tested. In this particular investigation, a cylindrical specimen that was 76 mm in radius and 64 mm in thickness was employed. Before the fibers and aggregates can be poured into the mold, the formwork must be meticulously cleaned and oiled on all interior surfaces. The frames were subsequently filled with coarse gravel and fibers, creating natural skeletons of aggregates (Figure 4a). At the very end, a cement grout with good flow properties was poured on top of the filled aggregate and fibers to fill in any remaining spaces between them (Figure 4b). Strong compaction or vibration was not needed at any step of the operation; the grouting was carried out using the gravity technique to fill the spaces between the aggregates and the fibers. Light compaction was performed on a specimen using a steel rod with a diameter of 6 mm to prevent honeycombing. After completing the first layer of PAFC, the GFM is next introduced with the required diameters and layers (Figure 4c). Afterward, the casting process is repeated to complete the second layer of the specimens. The appearance of the fabricated specimen after finishing is shown in Figure 4d.

## 3. Testing Setup

**Figure 5:**
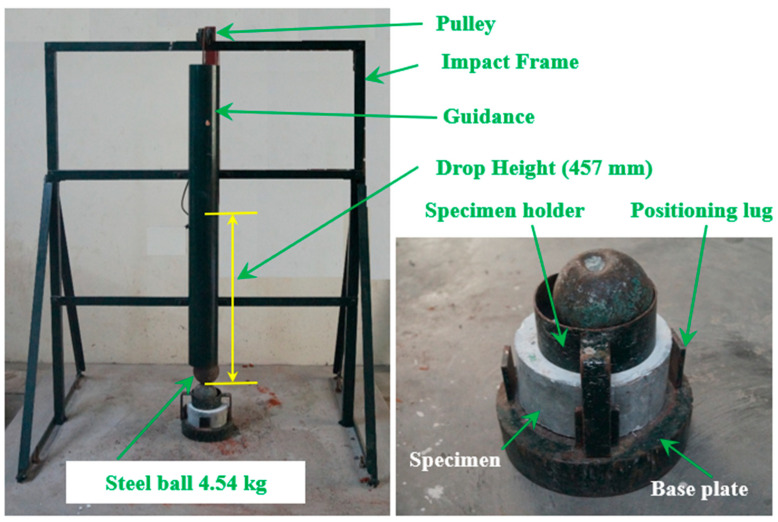
Impact test setup.

The 100 mm cubical specimens were cast to evaluate the compressive using the 300T compressive testing machine. PAFC’s impact strength was evaluated using a drop-weight impact test specified by the ACI Committee 544-2R [31]. Impact tests with drop weights are too simplistic since they do not need any load histories, deformations or vibrations. When conducting a test, the number of impacts required to generate first cracking (R1) and subsequent failure (R2) must be documented. The apparatus for determining the impact resistance of PAFC specimens is shown in Figure 5. This testing apparatus can drop a mass of 4.54 kg onto the target specimen from a height of 0.457 m. The raised steel ball strikes the ball of steel located on the specimen’s upper surface. The specimens were held in place and prevented from sliding horizontally by positioning lugs and a steel disc during the test. Both the R1 and R2 impact numbers were recorded. The failure is shown by the cracks that start on the specimen’s top surface and go down to the bottom surface of the specimen. The PAFC specimens that were visually evaluated indicated signs of a first crack and failure. The quantity of impact energy delivered by each hit is the same. The impact resistance is defined by the measured impact energy, computed by applying Equation (1).

Impact energy = *m* × *g* × *h* × *n* (1)

where *h* is height of drop steel ball, *m* is hammer mass, *n* is impact number and *g* is the gravity of acceleration (9.81 m/s^2^),

## 4. Results and Discussions

### 4.1. Strength of PAFC under Compression

As previously stated, 100 mm cubes were cast for both fibrous and non-fibrous specimens to test compressive strength. The recorded compressive strength of non-fibrous and fibrous specimens was 34.25% and 52.15%, respectively. Adding a 3% dosage of steel fibers exhibited a 67.05% improvement in compressive strength compared to non-fibrous specimens. The ability of uniformly distributed steel fibers to restrict macro-crack expansions is often the primary cause of an inherent improvement in compressive strength [32]. Crack propagation is delayed due to this process, reducing the stress concentration and even stress in concrete [33]. The bridging and strengthening of cracks are responsible for this action [34]. The behavior of PAFC under compression is consistent with the earlier studies [35,36,37]. Because of issues about workability and maintaining a uniform dispersion of fiber, the amount of fiber that may be used in PAFC is typically limited to 2%. Fiber balling occurs when the fiber dosage is more than 2%, which causes voids, lowers density and causes defects resulting in diminishing compressive strength [38,39]. Contrary to this, the PAFC manufacturing method eradicates these issues since mixed fibers and coarse aggregates were prepacked concurrently in the molds, and afterward, grout was injected to fill voids [40]. As a result, PAFC with a fiber content of 3% achieves excellent compressive strength.

### 4.2. Results and Discussion of Impact Strength

**Figure 6:**
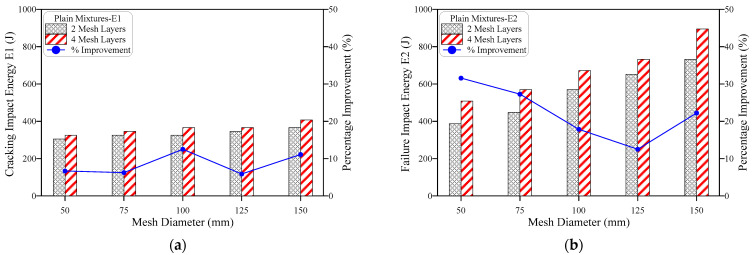
Effect of number of mesh layers on the impact energy of plain mixtures, (**a**) cracking energy E1, (**b**) failure energy E2.

**Figure 7:**
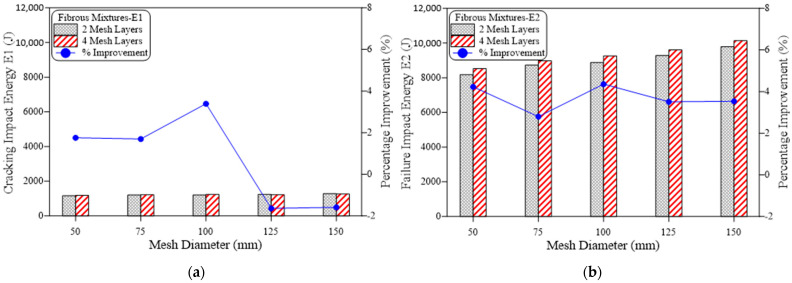
Effect of number of mesh layers on impact energy of fibrous mixtures, (**a**) cracking energy E1, (**b**) failure energy E2.

**Figure 8:**
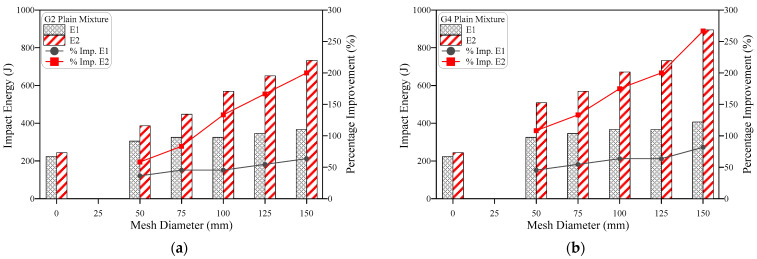
Effect of mesh diameter (ratio of reinforcement surface) on impact energy of plain mixtures, (**a**) 2-mesh layers, (**b**) 4-mesh layers.

**Figure 9:**
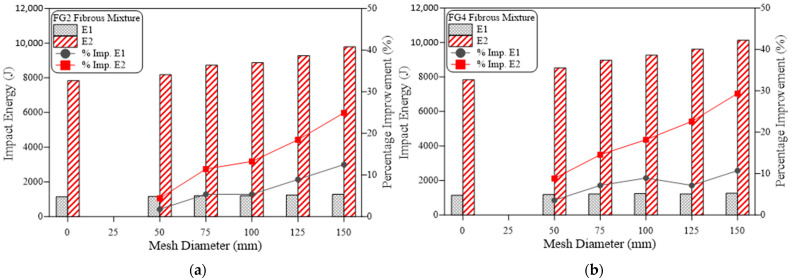
Effect of mesh diameter (ratio of reinforcement surface) on impact energy of fibrous mixtures, (**a**) 2-mesh layers, (**b**) 4-mesh layers.

**Figure 10:**
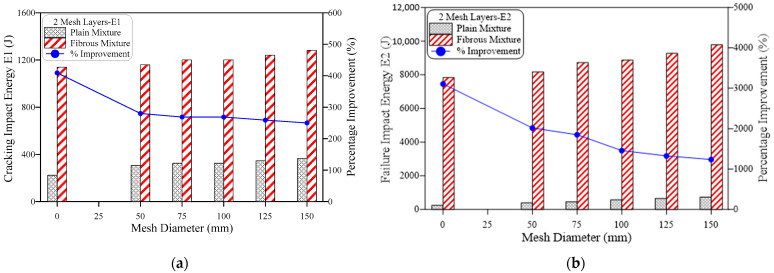
Effect of fiber on the impact energy of mixtures with 2-mesh layers, (**a**) cracking energy E1, (**b**) failure energy E2.

**Figure 11:**
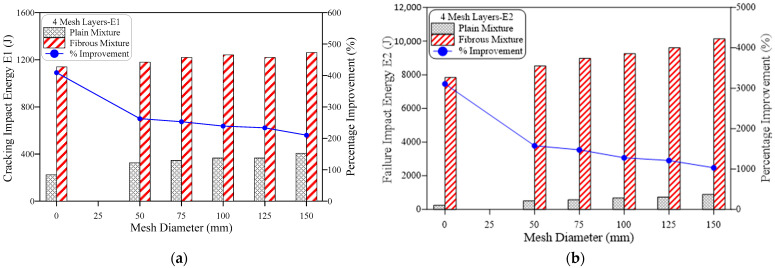
Effect of fiber on the impact energy of mixtures with 4-mesh layers, (**a**) cracking energy E1, (**b**) failure energy E2.

The obtained results of impact resistance at the cracking and failure stages from the ACI 544-2R repeated impact test are tabulated in Table 2 and depicted in Figure 6, Figure 7, Figure 8, Figure 9, Figure 10 and Figure 11. Three specimens were cast for each mixture and the average results were used for the discussions. It is observed from Table 2 that the standard deviation for the non-fibrous specimen varied from 1.0 to 2.08 for E1 and 1.53 to 4.04 for E2. For the fibrous specimens, the standard deviation values varied from 2.31 to 4.04 for E2 and 4.58 to 9.29 for E2. It is clear from a table that the calculated standard deviations are significantly less, which indicates fewer variations in test results. However, the standard deviation values for the non-fibrous specimens were less compared to the fibrous specimens. This phenomenon is due to the brittle nature of non-fibrous specimens. The obtained cracking impact numbers (R1) and failure impact numbers (R2) are presented in the figures in terms of the cracking impact energy (E1) and failure impact energy (E2), which are also listed in Table 2. The calculated impact energy represents the simple multiplication of the corresponding number of impact blows by the energy of a single drop-weight impact blow, as clarified in Equation (1). Thus, considering the drop weight and height recommended by the ACI 544-2R test, the impact energy of each single impact blow equals 20.345 J. Figure 6, Figure 7, Figure 8, Figure 9, Figure 10 and Figure 11 present the impact energy results in different ways to highlight the individual effects of the three investigated parameters, which are the number of the intermediate GFMs (2 or 4 meshes), the ratio of reinforcement surface to total sample surface (0.33, 0.5, 0.67, 0.83 and 1.0), and the effect of SF (0% and 3%). In the figures, the term “plain mixtures” was used to discuss the results of G2 and G4 mixtures that include no fibers, while the term “fibrous mixtures” was adopted for the mixtures that include 3% SF (FG2 and FG4).

The effect of increasing the number of GFM from two to four layers is depicted in Figure 6 for plain mixtures (G2 and G4), while Figure 7 shows this effect on the impact energy of fibrous mixtures (FG2 and FG4). Figure 6a,b show that the duplication of the number of GFM layers increased both the cracking impact energy E1 and the failure impact energy E2, regardless of the ratio of GFM reinforcement surface used, where it is obvious that the retained E1 and E2 of the specimens with four GFM layers were in general higher than those with two GFM layers. The percentage increase due to the duplication of the number of GFMs was in the range of 5.9 to 12.5% for E1, while it was in the range of 12.5 to 31.6% for E2 for all mesh diameters as shown in the figures. The main role of GFM is to work as an intermediate crack arrester that slows down the propagation of cracks among the contact area of the two layers of the specimen. Thus, its main function starts after the propagation of cracks among each layer, which explains the higher percentage of development at failure (E2) than at cracking (E1) [39,40,41]. Figure 7 shows that in most cases, the duplication of GFM led to an improvement in the retained impact energies of fibrous mixtures. However, this improvement is in general less than that of plain mixtures, where the maximum attained percentage development was 4.4%, as shown in Figure 7b. It is also clear in Figure 7 that higher percentage developments were obtained at the failure stage than at the cracking stage, which confirms the results obtained for plain mixtures. The lower development in fibrous specimens due to GFM duplication is attributed to the much larger influence of steel fibers on the impact response, so that the retained impact numbers of this group are much higher than plain mixtures. Hence, the additional numbers of blows gained by the duplication of GFM number are limited compared to the cracking and failure impact numbers of the two-layer fibrous specimens, which decreased the percentage development.

The effect of increasing the ratio of reinforcement surface of the GFMs from 0.33 to 0.5, 0.67, 0.83 and 1.0 on the retained impact energies is depicted in Figure 8 for plain mixtures and Figure 9 for fibrous mixtures. Figure 8 shows clearly that for all mixtures and at both cracking and failure stages, the increase of the GFM ratio of reinforcement surface increases the retained impact energy. This result is expected because decreasing the ratio of reinforcement surface of the GFM from 150 mm (the diameter of the test specimen) means that a partial contact intermediate area is covered by the GFM, which means that cracks can easily penetrate the intermediate zone crossing to the upper or lower layer. Hence, the shielding zone provided by the GFM decreases with the decrease of ratio of reinforcement surface, which in turn reduces its effect in decreasing the damage of the repeated impacts and vice versa. It is also noticed here that the percentage developments retained at the failure stage were higher than their corresponding percentage developments at the cracking stage, which is also attributed to the functionality of the GFM as discussed above. For the plain mixtures (Figure 8), the retained percentage developments in E1 for G2 and G4 mixtures (two and four layers of GFM) were in the ranges of 36.4 to 63.6% and 45.5 to 81.8%, respectively, while their corresponding percentage developments in E2 were in the ranges of 58.3 to 200% and 81.8 to 266.7%, respectively. Similar results were also obtained for the fibrous mixtures as depicted in Figure 9. It was also found that the developments in E2 were higher than E1, but the differences were not as high as those in the plain mixtures. Another thing to notice is that in general the retained percentage developments in the fibrous mixtures (FG2 and FG4) were lower than their corresponding plain mixtures (G2 and G4). This can also be attributed to the much higher recorded impact energies of the fibrous mixtures than plain mixture due to the existence of SF, which reduced the percentage of this development, as discussed earlier. The recorded percentage developments in E1 due to increasing the ratio of GFM reinforcement surface were in the ranges of 1.8 to 12.5% and 3.6 to 10.7% for FG2 and FG4 mixtures, respectively. On the other hand, the corresponding percentage developments in E2 for the same mixtures were in the ranges of 4.4 to 24.9% and 8.8 to 29.4%, respectively.

Figure 10 and Figure 11 show that steel fibers have a different level of effect on the impact performance compared to GFM, where using 3% of SF led to high improvements in E1 and huge jumps in E2 for all mixtures. The development of impact performance due to the incorporation of SF was slightly influenced by the number of incorporated GFMs and their ratio of reinforcement surface. As shown in the figures, the percentage development exhibited a trend of minor decrease with the increase of the GFM diameter (ratio of reinforcement surface). For instance, the percentage developments in E1 of the two GFM-layer mixtures (Figure 10a) due to SF incorporation were 280%, 268.8%, 268.8%, 258.8% and 250% for mixtures with ratio of GFM reinforcement surface of 0.33, 0.5, 0.67, 0.83 and 1.0, respectively. A similar trend of decrease was also recorded for E2 of this group (Figure 10b) and E1 and E2 of the four GFM layer, as shown in Figure 11. The decrease of the GFM diameter means that a part of the contact area between the two layers is not protected by GFM and steel fibers are penetrating through this area connecting the two layers. Hence, SF would share a larger percentage of the protection against cracking and fracturing, which means that the role of SF becomes more pronounced with the decrease of GFM. A confirmation of this explanation is that the highest percentage developments due to SF were obtained for specimens without GFM, as is clearly depicted in Figure 10 and Figure 11. For specimens with GFM, the percentage developments in E1 and E2 for the two GFM-layer and the four GFM-layer mixtures were in the ranges of approximately 210 to 280% and 1031 to 2016%, respectively, while the retained developments for the specimens without GFM were 409 and 3108%, respectively.

Two points should be discussed for the results shown in Figure 10 and Figure 11. The first is the superior percentage improvements obtained compared to GFM, while the second is the much higher developments obtained at failure stage (E2) compared to cracking stage (E1). Steel fibers are discrete reinforcement elements that spread along and across the matrix providing a multi-directional reinforcing action that slows down the evolution of the micro-cracks to wider and longer cracks, which are connected to compose the visible surface cracks [42,43]. Hence, SF provides a primary defence against excessive cracking under the repeated impact effect, which raised the retained E1 records significantly by more than 200%. After crack formation inside the matrix, the high tensile strength of the fibers allows sustaining the impact-induced tensile stresses across the crack that try to widen and open these cracks, while their hooked-end configuration enables them to fulfil this role owing to the adequate bond at the supporting ends on both sides of the crack. On the other hand, the GFM provides a localized shield on a specific area that limits its effect to this area, which explains the different roles and consequently the different percentage developments in the impact energy due SF and GFM. The function of SF becomes very obvious after crack formation and becomes greater with crack widening until the failure of the specimens [41], which explains the jumps in E2 of fibrous specimens compared to E1 and consequently the higher developments in E2. Several previous studies reported significant jumps in the impact performance of SF-reinforced mixtures compared to plain mixtures [44,45,46,47,48,49].

### 4.3. Impact Ductility

**Figure 12:**
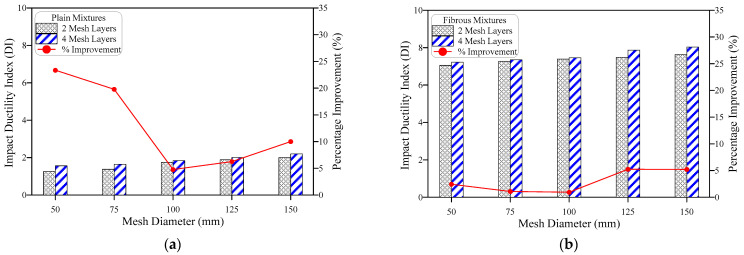
Effect of number of mesh layers on the impact ductility, (**a**) plain mixtures, (**b**) fibrous mixtures.

**Figure 13:**
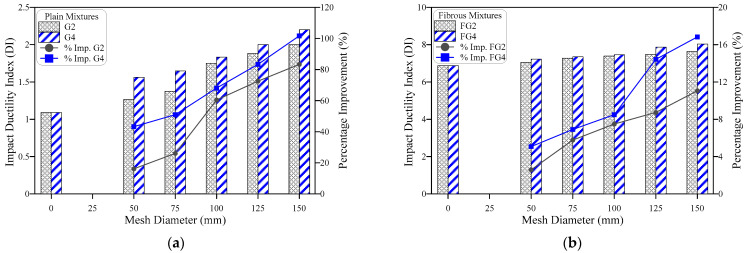
Effect of mesh diameter (ratio of reinforcement surface) on the impact ductility of (**a**) plain mixtures, (**b**) fibrous mixtures.

**Figure 14:**
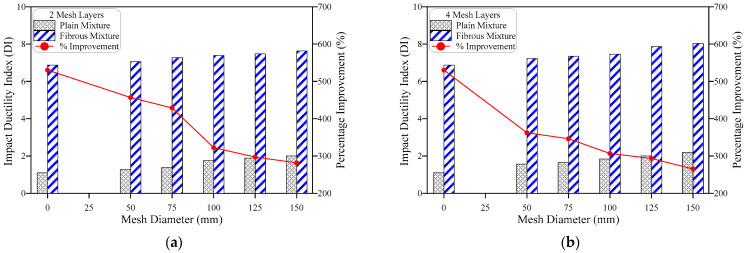
Effect of fiber on the impact ductility, (**a**) 2-mesh layers, (**b**) 4-mesh layers.

The term impact ductility index (DI) is used to express the ability of the concrete samples to absorb additional impact energy after the visual appearance of the first surface crack. Thus, the ductility index is defined as the ratio of the failure impact number to cracking impact number, which can similarly be expressed in terms of impact energy as E2/E1. Using this definition, the DI values were calculated for all mixtures and are presented in Table 2. Figure 12, Figure 13 and Figure 14 show the effect of the number of GFMs, the ratio of reinforcement surface (diameter) of GFM and SF on DI. Figure 12a shows that DI increases with the increase of the number of GFMs from 2 to 4 layers for plain mixtures, while Figure 12b explicitly reveals the same result for fibrous mixtures. The percentage DI improvements due to the number of mesh layers of both the groups were independent of the ratios of reinforcement surface of the GFM, which were in the range of 4.8 to 23.4% for plain mixtures and 0.9 to 5.2% for fibrous mixtures. As discussed in the previous section, the incorporation of GFM positively affects E2 more than E1, which explains the increase of DI with the increase of the number of GFM layers. On the other hand, as discussed earlier, the multidirectional influence of SF in crack arresting is much higher than the localized effect of GFM. Thus, the increased mesh layer number would result in smaller percentage developments for fibrous specimens that have already higher DI records compared to plain specimens.

Figure 13 shows that the increase ratio of GFM reinforcement surface increases the ductility index, which is expected as the coverage of the larger intermediate area between the two layers increases both E1 and E2. However, this increase is larger after crack formation, as discussed in Figure 8 and Figure 9, which means that DI would also be improved by increasing the ratio of reinforcement surface (diameter) of the meshes. As for the number of meshes and for the same reason, the effect of increasing the ratio of reinforcement surface of GFM on DI is more obvious for plain specimens (Figure 13a) than for fibrous specimens (Figure 13b). The percentage developments gained by increasing the ratio of reinforcement surface (diameter) of the GFM to 50, 75, 100, 125 and 150 mm were in the ranges of 16.1 to 83.3% and 43.2 to 101.7% for G2 and G4 plain mixtures, while they were in the ranges of 2.6 to 11.1% and 5.1 to 16.8% for FG2 and FG4 fibrous mixtures, respectively.

Steel fibers have the greatest effect on the impact performance compared to the other investigated parameters, and, as discussed earlier, the incorporation of SF led to great jumps in E2 while it led to lower developments in E1. Thus, the ratio of E2 to E1 increased significantly, leading to the high differences in DI between plain mixtures and fibrous mixtures shown in Figure 14. As shown in the figure, the DI records of all plain mixtures ranged from 1.1 to 2.2, while those of fibrous mixtures ranged from 6.9 to 8.0. Hence, DI records of fibrous mixtures were duplicated several times due the positive activity of SF. The percentage developments in DI of fibrous specimens compared to their corresponding plain ones were in the range of 265 to 530%. According to Murali et al. [35], the ductility index for the steel fiber-based PAFC beams varied from 2.53 to 2.80. Vatin et al. [6] reported that the ductility index PAFC comprising steel fibers and asphalt-coated aggregate varied from 5.3 to 5.8. This phenomenon is attributed to the combined action of asphalt-coated aggregate and steel fibers leading to an increase in the impact ductility index. The observed ductility index of steel fiber-based specimens are in good alignment with the earlier literature [50,51].

### 4.4. Failure Pattern

**Figure 15:**
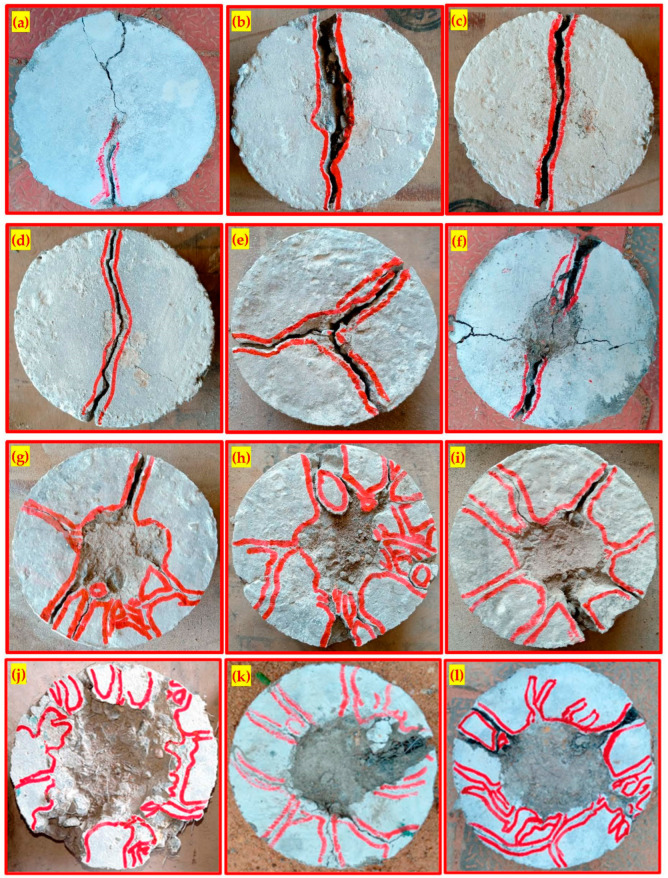
Failure pattern of PAFC. (**a**) CC, (**b**) G2-50, (**c**) G2-75, (**d**) G2-100, (**e**) G2-125, (**f**) G2-150, (**g**) FC, (**h**) FG2-50, (**i**) FG2-75, (**j**) FG2-100, (**k**) FG2-125 and (**l**) FG2-150.

Figure 15 depicts the failure pattern of plain specimens with GFM and steel fiber specimens with GFM failure patterns under impact loading. The impact resistance of the plain specimens without GFM was substantially lower. When the first crack appeared, the specimens immediately failed, demonstrating their brittle nature when subjected to impact loads. Figure 15a–f depicts the diagonal line crack that occurred when the fracturing limit was achieved. Following a few impacts, this crack widened until it hit the edges of the cylinder, continued down to the bottom surface and eventually split the specimen in half. According to the vast majority of the relevant published research, this is the most often seen instance of brittle failure in plain cylinders with and without GFM [52,53,54]. On the other hand, it was also observed that there was a possibility of a small fracture forming before the collapse of the specimen. It is also important to note that just a small percentage of the specimens failed in a brittle manner, but those that did were broken into three/four separate pieces. The brittle tendency may be linked to the concrete, which is brittle in nature and lacks any bridging materials that retain the bonding along the two ends of the developed crack. It is anticipated that strains will move horizontally in all locations when the impact force is carried circularly from the steel ball to the top surface of specimens. Because of the heterogeneous structure of concrete, cracks may appear in any orientation, some of which may be more susceptible to failure than others. As a direct consequence, there is a possibility that not one but several cracks may occur and spread in any diagonal direction.

The impact strength capability of fibrous specimens and GFM displayed unique qualities, which were significantly enhanced owing to the addition of fibers. A center spherical fracturing area was generated underneath the steel ball due to the greater number of impact strikes that were absorbed (Figure 15g–l). This zone grew in size as the number of impacts increased, causing the surface to fracture, revealing small-scale surface cracks. Figure 15g–l depicts the surface failure of the specimen comprising steel fiber and GFM. As the number of impacts continued, the micro-cracks were widened and turned to macro-cracks, propagated towards the outside perimeter and spread to the specimens’ bottom surface. As shown in Figure 15k, the fibers continue to bridge the two sides of the cracks, even though the link between the matrix and fibers has progressively broken down, which ultimately led to the breakdown of the specimens due to fiber pullout. It is important to highlight that the presence of GFM delays the crack propagation when traveling from top to bottom of the specimens. Layers of GFM in PAFC serve as a barrier, interrupt the rapid crack growth and extend the duration of the failure with higher impact energy capacity. This is the most common ductile failure in fibrous concrete cylinders and is well aligned with earlier research [55,56].

### 4.5. Fiber Oreintation

**Figure 16:**
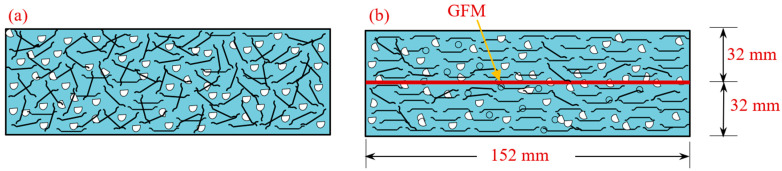
Comparison of fiber orientation, (**a**) 3D orientation in FC, (**b**) Planar orienatation in FG2-150.

Two-layered PAFC exhibited a greater impact resistance due to the fiber alignments. It is possible to hypothesize that this phenomenon is brought about by two different processes: the fibers’ alignment and the fiber matrix’s bonding action. One layer of FC specimens has 3D fiber orientation, whereas the two-layered PAFC specimens have planar fiber alignment (Figure 16). It is reported that the specimen with planar fiber alignment exhibited excellent impact resistance and these findings are supported by earlier research [36,39,57].

### 4.6. Failure Mechanism

Under impact loading, the most common mechanisms for PAFC fracture are cracking, shearing and compaction. Compression, tension or restricting pressures may cause the concrete to fracture in the direction of the forces exerted. Nevertheless, the production of eventual fractures in the complete concrete specimen depends on the concrete’s essential properties, which include fibers and GFM. Other findings imply that when the first diagonal crack appears, a large percentage of the initial kinetic energy is transferred from the concrete to the fibers.

**Figure 17:**
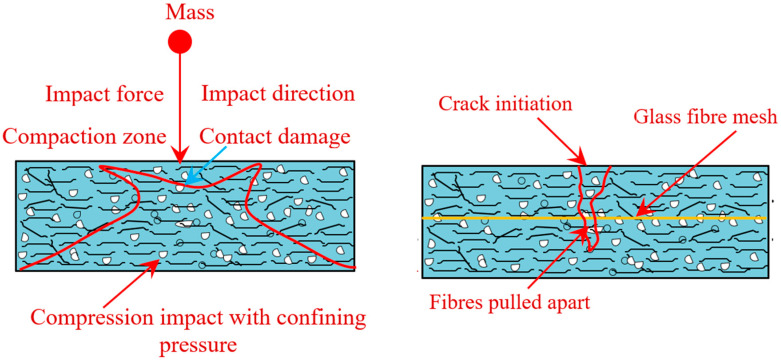
Failure mechanism of PAFC under impact loading.

Consequently, after the first fracture has developed, the fibers can still bridge it and disperse the energies to multiple adjacent areas inside the concrete. Concrete is damaged when the fibers can no longer inhibit cracks from occurring and are pulled out by the impact stress distribution [32]. Contact damage, fiber rupturing, matrix failure and fiber delamination occurred when specimens were exposed to impact testing (Figure 17). There was damage to the specimen due to direct collision. Compressive bending on the impact plane ultimately causes the matrix fibers to fail [58]. Finally, fiber debonding advances to neighboring locations due to tensile bending at the bottom surface. Fibers are separated from their matrix in a critical phase in the failure process, which severely influences the material’s strength [59].

## 5. Conclusions

The combined action of steel fibers and glass fiber mesh on preplaced aggregate concrete was investigated against the drop weight impact. Five different ratios of reinforcement surface (0.33, 0.5, 0.67, 0.83 and 1.0) and two and four layers of GFM are sandwiched between the concrete. The effect of GFM and the combined action of GFM and steel fiber were also investigated. Based on the findings obtained from the research, the following conclusions may be inferred.

1. The addition of a 3% dosage of steel fiber in PAFC revealed a compressive strength that was improved by 67.05% compared to non-fibrous specimens. The capability of evenly dispersed steel fibers to inhibit macro-crack expansions is frequently the primary reason for an inherent improvement in compressive strength. The PAFC casting technique eradicates the workability issues and fiber clustering due to the higher fiber content (more than 2%), since fibers and aggregates are pre-packed into the mold followed by grouting.
2. Duplicating the number of intermediate GFMs from two to four increased the retained E1 and E2 of plain and fibrous mixtures. The percentage development in the impact energy due to this duplication was independent of the GFM ratio of reinforcement surface (diameter), while it was higher at failure stage (E2) compared to cracking stage (E1). As the main function of GFM is to arrest cracks from propagating across the contact area between the two specimen’s layers, its main function starts after cracking, which explains the higher developments in E2 compared to E1. For instance, the percentage developments in E1 of plain mixtures were in the range of 5.8 to 12.5%, while those of E2 ranged from 12.5 to 31.6%.
3. GFM ratio of reinforcement surface (diameter) has a noticeable effect on the recorded impact energies, where decreasing the GFM ratio of reinforcement surface from the full intermediate contact area (150 mm diameter) decreased both E1 and E2, where the percentage improvement in E1 and E2 was increased with the increase of GFM ratio of reinforcement surface from 0.33 up to 150 mm. Due to the role of GFM in the specimens, this effect was also more pronounced at the failure stage than at the cracking stage, where the percentage developments (compared to reference specimens without GFM) in E1 of plain mixtures ranged from 36.4 to 81.8%, while the corresponding developments in E2 were in the range of 58.3 to 266.7%.
4. Steel fibers are significantly superior to GFM in enhancing the impact performance. Comparing the impact energies of the fibrous specimens incorporating 3% SF with their corresponding plain (0% SF) specimens, significant improvements of 210 to 409% were recorded for E1, while great jumps of 1032 to 3108% were recorded for E2. These percentage improvements were much higher than those obtained by utilizing the GFM, which is attributed to the mechanisms of each mesh being a crack arresting material, where SFs are distributed across the whole volume in different angles composing a multi-directional discrete reinforcing matrix, while GFM provides a localized shield over only the contact area between the two layers of the specimen.
5. Because the effect of GFM number, GFM ratio of reinforcement surface and SF are higher at the failure stage (E2) than at the cracking stage (E1), the ductility index (DI) increased due to their increase. As the effect of SF was superior to that of GFM, DI records of fibrous specimens were much higher than those of plain specimens. The ductility indices of the plain specimens were in the range of 1.1 to 2.2, while those of fibrous specimens ranged from 6.9 to 8.0. The percentage developments in DI due to increasing the number of layers and the ratio of reinforcement surface of GFM was much higher for plain specimens than for fibrous specimens due to the high DI records of fibrous specimens. Increasing the ratio of reinforcement surface of GFM improved DI of plain mixtures by 16.1 to 101.7%, while this improvement was 2.6 to 16.8% for fibrous mixtures. Similarly, duplicating the number of GFMs increased DI of plain mixtures by 4.8 to 23.4% and DI of fibrous mixtures by 0.9 to 5.2%, while the percentage developments in DI due to the incorporation of 3% of SF were in the range of 265 to 530%.
6. The impact resistance was much lower in GFM-based non-fibrous specimens, irrespective of GFM layers. These specimens failed as soon as a fracture emerged, revealing their brittleness. Fibrous specimens with GFM have distinct impact strength capabilities that were significantly boosted by including fibers indicating ductile failure. Moreover, the layers of GFM in PAFC function as a barrier, prevent quick crack propagation and lengthen the duration of the failure with a more significant impact energy absorption capacity.
7. Although the PAFC poses an excellent impact strength and there are some limitations, including: the dosage of steel fiber in PAFC is restricted to 6%; the number of GFM layers is restricted to four to avoid the shearing effect of PAFC under impact loading.

The effect of GFM ratio of reinforcement surface and the number of GFM layers in PAFC on the impact strength was examined. The other type of mesh insertion with varying depth of specimen, hybrid mesh insertion, the effect of drop weight and height of hammer on the different types of fibers and new composites (geopolymer concrete or sustainable concrete, recycled aggregate concrete) could be the scope for future studies.

## Figures and Tables

**Table 1:** Mixing combinations.

Mixture Id	*c*/*s*	*w*/*c*	Diameter of GFM (mm)	Ratio of Reinforcement	Number of GFM Layer	Fiber Dosage (%)	SP (%)
CC	1.0	0.45	0	0	0	0	0.4
G2-50	1.0	0.45	50	0.33	2	0	0.4
G2-75	1.0	0.45	75	0.5	2	0	0.4
G2-100	1.0	0.45	100	0.67	2	0	0.4
G2-125	1.0	0.45	125	0.83	2	0	0.4
G2-150	1.0	0.45	150	1.0	2	0	0.4
G4-50	1.0	0.45	50	0.33	4	0	0.4
G4-75	1.0	0.45	75	0.5	4	0	0.4
G4-100	1.0	0.45	100	0.67	4	0	0.4
G4-125	1.0	0.45	125	0.83	4	0	0.4
G4-150	1.0	0.45	150	1.0	4	0	0.4
FC	1.0	0.45	0	0	0	3	0.6
FG2-50	1.0	0.45	50	0.33	2	3	0.6
FG2-75	1.0	0.45	75	0.5	2	3	0.6
FG2-100	1.0	0.45	100	0.67	2	3	0.6
FG2-125	1.0	0.45	125	0.83	2	3	0.6
FG2-150	1.0	0.45	150	1.0	2	3	0.6
FG4-50	1.0	0.45	50	0.33	4	3	0.6
FG4-75	1.0	0.45	75	0.5	4	3	0.6
FG4-100	1.0	0.45	100	0.67	4	3	0.6
FG4-125	1.0	0.45	125	0.83	4	3	0.6
FG4-150	1.0	0.45	150	1.0	4	3	0.6

The ratio of reinforcement is obtained from the area of reinforcement (GFM) to the total area of the specimens (area of 150 mm diameter specimen).

**Table 2:** Impact test results.

Mix Id	Impact Numbers		Impact Energies (J)		Standard Deviation		Ductility Index (DI)
	R1	R2	E1	E2	E1	E2	
CC	11	12	223.8	244.1	1.00	1.53	1.09
G2-50	15	19	305.2	386.6	2.08	2.08	1.27
G2-75	16	22	325.5	447.6	1.15	2.08	1.38
G2-100	16	28	325.5	569.7	1.53	2.00	1.75
G2-125	17	32	345.9	651.0	1.53	2.08	1.88
G2-150	18	36	366.2	732.4	0.58	2.52	2.00
G4-50	16	25	325.5	508.6	2.08	2.08	1.56
G4-75	17	28	345.9	569.7	1.73	2.65	1.65
G4-100	18	33	366.2	671.4	1.15	2.89	1.83
G4-125	18	36	366.2	732.4	2.00	4.04	2.00
G4-150	20	44	406.9	895.2	2.08	3.61	2.20
FC	56	385	1139.3	7832.8	3.79	4.36	6.88
FG2-50	57	402	1159.7	8178.7	3.51	8.19	7.05
FG2-75	59	429	1200.4	8728.0	3.06	9.02	7.27
FG2-100	59	436	1200.4	8870.4	4.04	9.29	7.39
FG2-125	61	456	1241.0	9277.3	3.06	4.58	7.48
FG2-150	63	481	1281.7	9785.9	3.79	8.14	7.63
FG4-50	58	419	1180.0	8524.6	3.51	8.50	7.22
FG4-75	60	441	1220.7	8972.1	3.00	8.33	7.35
FG4-100	61	455	1241.0	9257.0	2.65	6.00	7.46
FG4-125	60	472	1220.7	9602.8	3.21	7.51	7.87
FG4-150	62	498	1261.4	10,131.8	2.31	7.55	8.03

## Data Availability

Not applicable.
